# New Year Address of *Zoological Research*

**DOI:** 10.13918/j.issn.2095-8137.2017.001

**Published:** 2017-01-18

The year 2016 was a wonderful and important one for *Zoological Research* (*ZR*). In recognition of its impressive progress and great potential to develop into a leading journal in the field, *ZR* is now supported by the "Project for Enhancing International Impact of China STM Journals" (PIIJ) (Class B) (2016-2018). Having wide coverage, PIIJ is the most influential project in China for supporting and promoting the development of English language academic journal publications. In addition, *ZR* has achieved the "International Impact Academic Journal of China" title five years in a row. 

The success of *ZR* depends on its unique niche in the field and the joint effort of its strong editorial team. Emphasizing the scope of *ZR*, namely, "Primates and Animal Models", "Conservation & Utilization of Animal Resources", and "Animal Diversity and Evolution", two special issues were released in 2016: "*The Amphibian and Reptile Biodiversity of Qinghai-Tibetan Plateau*" and "*CRISPR/Cas9, Model Animals and Human Diseases*". Moreover, to promote communication and collaboration among peer researchers, *ZR* is hosting the "2017 Frontiers in Zoology Symposium" in February 2017. 

To further strengthen the editorial board and widen its scope of expertise, *ZR* continues to recruit local and overseas scientists to serve on its board. In 2016, *ZR* engaged four renowned scientists with varied expertise to join the vibrant *ZR* editorial team. These new board members include: Dr. Wai-Yee Chan, Chair Professor and Director, School of Biomedical Sciences, Chinese Hong Kong University, Hong Kong SAR, China; Dr. LeAnn Blomberg, Beltsville Agricultural Research Center (BARC), United States Department of Agriculture, USA; Dr. Hua-Xin (Larry) Liao, Professor, College of Life Science and Technology, Jinan University, China, and Professor, Duke University, USA; and Dr. Meng-Ji Lu, Professor, Institute of Virology, University of Duisburg-Essen, Germany. In addition, Dr. Guojie Zhang, a well-known expert on animal genomics who holds a position at the China National GeneBank, BGI-Shenzhen, and the Department of Biology, University of Copenhagen, as well as a guest professorship at the Kunming Institute of Zoology, Chinese Academy of Sciences, will also join the editorial board. With these new members, the editorial board of *ZR* now includes 48 distinguished scientists with expertise across multiple disciplines. 

To further its scientific reach, *ZR* has continued to promote and market the journal during this past year. A new and more interactive homepage was launched and, as an open access journal, full-text articles can be downloaded via the homepage, PubMed Central, and SciEngine. To expand our readership, abstracts of published articles are also available online in Chinese. Information regarding the new initiatives and measures of *ZR* will continue to be shared with our readers through our official WeChat account (ZoolRes). In addition, *ZR* has now been indexed by Scopus and other related databases, and has a CitesScore value of 0. 48 and ranks 786 among 1 549 journals in the subject "Medicine". 

*ZR* will continue to publish innovative zoological studies and advance knowledge in scientific research. With the proud tradition of attracting outstanding scholars with diverse backgrounds and expertise to write for the journal, as well as the hard work and dedication of our editors and editorial board members, *ZR* has become a representative international scientific journal in animal studies. With a growing number of international submissions and article citations, as well as an increasing impact factor, we believe that *ZR* will confidently march into its thirty-seventh year and accomplish its goal of becoming an iconic journal in animal research. *ZR* could not have achieved what it has without the support of you, our readers. It is a journal that belongs to all members of the animal research community, and we look forward to continuing to work with you all to nurture and carry *ZR* to the next stage in 2017. 

Sincerely


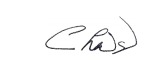


Wai-Yee Chan, Executive Editor-in-Chief *School of Biomedical Sciences*, *The Chinese University of Hong Kong, China*


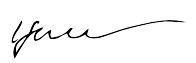


Yong-Gang Yao, Editor-in-Chief *Kunming Institute of Zoology*, *Chinese* *Academy of Sciences, Kunming 650223, China*

